# Efficacy and Safety of Wenxin Granules and Propafenone in Treatment of Atrial Premature Beats: A Systematic Review and Meta-Analysis

**DOI:** 10.1155/2020/3961091

**Published:** 2020-07-23

**Authors:** Yi Yuan, Xing-jiang Xiong, Duo-duo Li, Hai-xia Li, Jian-ping Fu, Hong-li Wu

**Affiliations:** ^1^Beijing University of Chinese Medicine, Beijing 100029, China; ^2^Guang' Anmen Hospital, China Academy of Chinese Medical Sciences, Beijing 100053, China; ^3^Dongzhimen Hospital, Beijing University of Chinese Medicine, Beijing 100700, China; ^4^Institute of Basic Research in Clinical Medicine, China Academy of Chinese Medical Sciences, Beijing 100700, China

## Abstract

**Objective:**

A meta-analysis was conducted on the clinical efficacy and safety of Wenxin granules and propafenone for the therapy of atrial premature beats (APBs).

**Methods:**

A randomized controlled trial (RCT) of Wenxin granules and propafenone in the therapy of APB was systematically searched until June 1, 2019. Meta-analysis was conducted with review manager (RevMan) 5.3. For the evaluation of methodological quality for randomized controlled trials, the Cochrane tool was used to assess the risk of bias. For the evaluation of the evidence quality, the online GRADEpro GDT was used.

**Results:**

Eleven RCTs with 1149 participants were included in this study. It has been identified that Wenxin granules combined with propafenone have better clinical efficacy than the use of propafenone alone in the treatment of APB (OR = 3.89, 95% CI (2.03, 7.44), *P* < 0.0001, low-dose propafenone; OR = 4.24, 95% CI (1.32, 13.60), *P* = 0.02, high-dose propafenone). There is no difference in clinical efficacy between the Wenxin granules alone and high-dose propafenone in the treatment of APB (OR = 1.17, 95% CI (0.65, 2.11), *P* = 0.60), and Wenxin granules alone are superior to the low-dose propafenone in the treatment of APB (OR = 2.56, 95% CI (1.34, 4.89), *P* = 0.004). Wenxin granules combined with propafenone can reduce the incidence of sinus bradycardia caused by propafenone (OR = 0.15, 95% CI (0.03, 0.70), *P* = 0.02). There was no significant difference between Wenxin granules combined with propafenone and propafenone alone in causing the atrioventricular block, dizziness, xerostomia, gastrointestinal symptoms, and tongue paresthesia. There was no significant difference between Wenxin granules alone and propafenone alone in causing dizziness, xerostomia, gastrointestinal symptoms, tongue paresthesia, frequent premature ventricular contractions, and prolongation of R-R interval.

**Conclusion:**

Very low-quality evidence showed that Wenxin granules may be superior to low-dose propafenone in the treatment of APB. Wenxin granules may reduce the incidence of sinus bradycardia caused by propafenone. Limited by the quality of included RCTs, the conclusions of this study still need further verification.

## 1. Introduction

Atrial premature beats (APBs) can occur in any part of the atrium and are found in more than 60% of healthy adults. The incidence of atrial premature beats is higher in patients with organic heart diseases and is more common in patients with atrial lesions, atrial enlargement, and heart failure [1]. Most atrial premature beats do not need urgent treatment. APB usually has a good prognosis and little influence on hemodynamics [2]. However, the frequent occurrence of APB has a coupling; in particular, when APB is not transmitted down, cardiac output can be significantly reduced. The more important significance of APB is to trigger other more serious arrhythmias, such as reentrant supraventricular tachycardia and atrial tachycardia-induced by APB, atrial flutter, and atrial fibrillation. At this time, it will have a more severe impact on hemodynamics, affect left ventricular systolic and diastolic functions, and induce heart failure and pulmonary hypertension [3]. Propafenone, as a broad-spectrum antiarrhythmic drug, has the advantages of quick effect and lasting effect and is widely used in clinical treatment, but large-dose use also increases the incidence of adverse reactions [4].

Traditional Chinese Medicine holds that atrial premature beats belong to the categories of “Fright” and “Severe Palpitation.” The mild cases are “Fright” and the severe cases are “Severe Palpitation.” It is mainly caused by emotional injury, deficiency of body constitution, and invasion of external pathogens which are cause and effect of each other and influence each other, leading to Qi and Blood deficiency, imbalance of Yin and Yang, and disorder of heart governing blood vessels. Therefore, the treatment starts with regulating the ups and downs of Yin and Yang and dredging the stasis of Qi and Blood [5]. Wenxin granules are a new generation of antiarrhythmic Traditional Chinese Medicine compounds developed in China and have a clinical curative effect on arrhythmia [6]. The granules comprise five constituent parts: Rhizoma Nardostachyos, Lanceolata, Panax Notoginseng, Amber, and Rhizoma Polygonatum [7]. Tian et al. [8] had shown that Wenxin granules had protective effects on myocardium and arrhythmia. Wenxin granules could inhibit the inflammatory response, reduced oxidative stress, regulated vasomotor dysfunction, reduced apoptosis, and protected endothelial cells from injury, myocardial ischemia, fibrosis, and hypertrophy. In this study, the clinical efficacy and safety of Wenxin granules and propafenone for the therapy of atrial premature beats were evaluated by literature retrieval and meta-analysis method, so as to provide a reference for clinicians to optimize the treatment scheme.

## 2. Methods

The systematic review protocol was registered at PROSPERO (NO: CRD42020148712). Registration details are available at https://www.crd.york.ac.uk/PROSPERO/.

### 2.1. Type of Study

RCTs that assessed the effects of Wenxin granules and propafenone in the therapy of APB were included. The language was limited to Chinese and English.

### 2.2. Type of Participants

Diagnostic criteria in line with arrhythmias (atrial premature beats), regardless of gender, race, and age, with or without organic heart disease will be included. All patients fulfilled the ACC/AHA/ESC guidelines for the management of patients with supraventricular arrhythmias [9] and guidelines for diagnosis and treatment of common internal diseases in Chinese Medicine Symptoms in Chinese Medicine [10].

### 2.3. Type of Interventions

Three specific comparisons including Wenxin granules will be taken into account: Wenxin granules, Wenxin granules compared with low-dose propafenone, and Wenxin granules compared with high-dose propafenone.

The control intervention included low-dose propafenone and high-dose propafenone (same dose as propafenone in the experimental group).

See Table 1 for the specific composition of Wenxin granules.

### 2.4. Type of Outcome

Any of the following outcome indicators had been reported in clinical studies.

#### 2.4.1. Primary Outcomes

Clinical efficiency: changes in the number of premature beats before and after treatment.

#### 2.4.2. Security Index

Adverse reaction rate: specific adverse reactions reported, and the number of cases reported.

### 2.5. Search Strategy

We would search the following databases with no restriction on publication status or language: PubMed, EMBASE, The Cochrane Library, China National Knowledge Infrastructure (CNKI), Chinese VIP Information (VIP), and WanFang Data. In addition, we retrieved ongoing clinical trials from The WHO ICTRP Search Portal.  Table 2 outlines the detailed search strategy of PubMed and CNKI.

### 2.6. Study Selection and Data Extraction

Two evaluators (Y. Yuan and D. D. Li) independently screened and extracted literature data according to inclusion and exclusion criteria, cross-checked, discussed, or submitted to a third party (X J. Xiong) in case of disagreement. EndNote X9 was used to manage and screen the literature, and a self-made data extraction table was used to extract the data. The basic information of the included research was mainly included, such as the first author and publication time; the basic characteristics of the patients were included, such as sample size, age, and disease classification and time; research design type and methodology characteristics, outcome indicators, and outcome measurements of concern were also included. In addition, the authors would be contacted in consideration of errors in the included clinical studies or any lack of detail in the studies. If the author did not reply, a consensus would be reached on the basis of the available information.

### 2.7. Quality Assessment

Two reviewers (Y. Yuan and D. D. Li) would use the Cochrane Collaboration tool to assess the risk of bias for included studies as follows: random sequence generation (selection bias), allocation concealment (selection bias), blinding (performance bias and detection bias), incomplete outcome data (attrition bias), selective outcome reporting (reporting bias), and others [11]. Each entry represents a characteristic of the included research. The estimate for each clause involves evaluating the risk of bias as “low risk,” “high risk,” and “unclear.” In all researches, if most of the information was from the research that had a low risk of bias, then the existence of bias would be unlikely to seriously influence the consequences of the study; if most of the material was from the low risk of bias or bias risk uncertainty research, this would explain the existence of bias caused by the result of the research of doubt; if the ratio of information was found on the high bias risk research enough to influence the explanation of the consequences, then the existence of bias would severely reduce the credibility of the results of the research. We evaluated the risk of bias using the “bias risk summary graph,” which described the proportion of studies (low risk, high risk, and risk uncertainty) for each item in the tool.

### 2.8. Data Analysis

We would conduct a statistical analysis using RevMan (version 5.3. Copenhagen: The Nordic Cochrane Centre, The Cochrane Collaboration, 2014). Standardized mean difference (SMD) with 95% confidence intervals (CIs) would be used to analyze continuous data and odds ratios (OR) with 95% CI for dichotomous data. The initial subgroup setting would be made in accordance with the outcomes and interventions. The heterogeneity of every group/subgroup would be evaluated by using both the *X*^2^ test and the *I*^2^ statistic. An *I*^2^ value higher than 50% would be considered to be indicative of significant heterogeneity, and further analysis including sensitivity and subgroup analysis would be conducted, in order to explore the possible sources of heterogeneity.

If high levels of heterogeneity (*I*^2^ > 75%) were still detected after exploration, which indicates considerable heterogeneity, we would simply carry out descriptive analyses. At the other end of the spectrum, we would perform a fixed-effect (*I*^2^ < 50%) or random-effects (75% > *I*^2^ >50%) meta-analysis [11].

Sensitivity analysis was the reanalysis of meta-analysis to replace random or unclear alternative decisions or the value range of decisions. When the sensitivity analysis showed that the overall results and conclusions were not affected by different decisions that may be made during the system evaluation, the results of the system evaluation could be considered to have a higher degree of affirmation. When sensitivity analysis identified specific decisions or missing information that could significantly affect the outcome of the system evaluation, additional resources could be used to try and resolve uncertainty and obtain additional information (perhaps by contacting trial authors and obtaining individual patient data). Funnel chart was a simple scatter plot and reflected the research to certain sample size or the intervention effect of estimate accuracy under single study, generally when the meta-analysis included in at least ten researches could use the funnel chart asymmetric inspection because if too few studies were included, the inspection efficiency would be low and would not be any difference between real opportunities and asymmetry. GRADE divides the quality of evidence into four levels: high, medium, low, and very low. The risk of bias, inconsistencies, imprecision, indirectness, and publication bias of research could reduce the quality of evidence.

## 3. Results

### 3.1. Description of Studies

**Figure 1:**
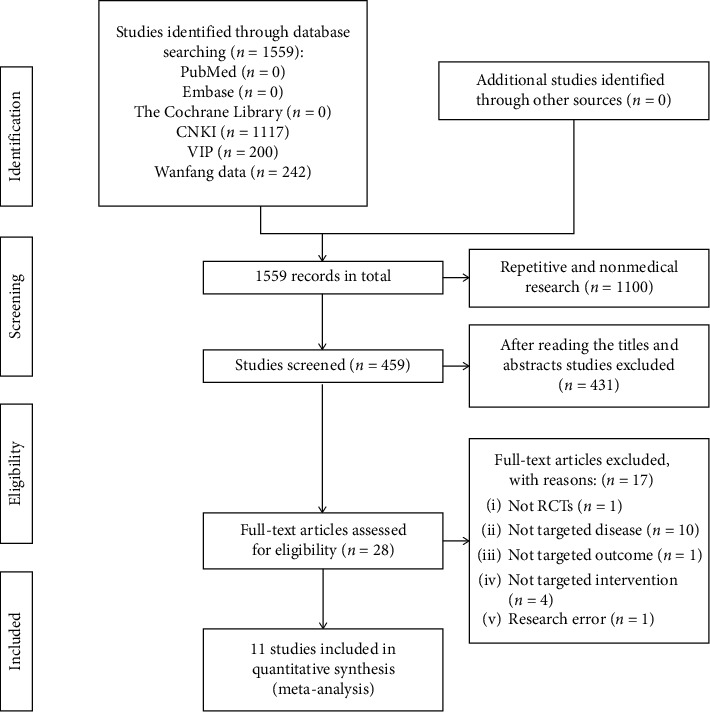
Flow chart of study selection.

1559 relevant studies were initially detected, and 1,100 duplicate studies were excluded. After 431 articles were excluded on the basis of the inclusion and exclusion criteria, 28 full-text articles were further read, and 11 randomized controlled trials that met the inclusion criteria were finally included. The retrieval and screening flow chart is shown in Figure 1.

### 3.2. Study Characteristics

The basic characteristics of the study are shown in Table 3. Eleven RCTs [4, 5, 12–20] with a total of 1149 participants with APB were included in this review.

All the included articles were in Chinese. A total of five comparisons were made among the 11 included studies. Three articles compared propafenone 100 mg and Wenxin granules with propafenone 100 mg. Two articles compared propafenone 150 mg and Wenxin granules with propafenone 150 mg. Three articles compared Wenxin granules with propafenone 150 mg. Two articles compared Wenxin granules with propafenone 100 mg. One article compared Wenxin granules with propafenone 100–150 mg.

### 3.3. Risk of Bias of Included Trials

**Figure 2:**
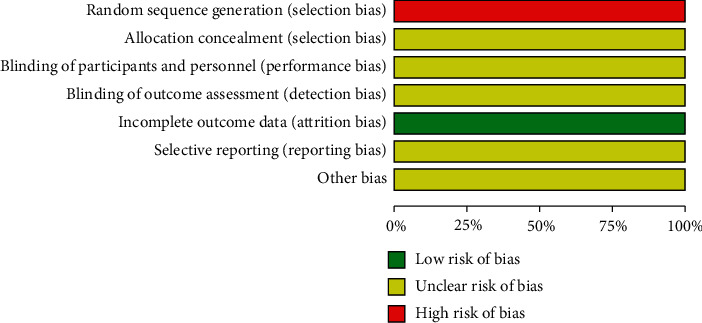
Risk of bias graph.

Specific randomized methods were not mentioned in all the studies, and due to limited information, it was impossible to determine the low or high risk of these studies. The risk of bias in the included study is shown in Figure 2.

### 3.4. Clinical Efficacy

#### 3.4.1. Comparison 1: Propafenone 100 mg and Wenxin Granules with Propafenone 100 mg

Three trials [4, 5, 13] were involved in this comparison; there was no statistical heterogeneity among the studies (*I*^2^ = 0%, *P* = 0.81). Therefore, the fixed-effect model was adopted for meta-analysis. Meta-analysis results of the fixed-effect model showed that the combination group was better than the propafenone group. The difference was statistically significant (OR = 3.89, 95% CI (2.03, 7.44), *P* < 0.0001).

#### 3.4.2. Comparison 2: Propafenone 150 mg and Wenxin Granules with Propafenone 150 mg

Two trials [12, 14] were involved in this comparison; there was no statistical heterogeneity among the studies (*I*^2^ = 0%, *P*=0.85). Therefore, the fixed-effect model was adopted for meta-analysis. The meta-analysis results of the fixed-effect model showed that the combination group was better than the propafenone group. The difference was statistically significant (OR = 4.24, 95% CI (1.32, 13.60), *P*=0.02).

#### 3.4.3. Comparison 3: Wenxin Granules with Propafenone 150 mg

Three trials [15–17] were involved in this comparison; there was no statistical heterogeneity among the studies (*I*^2^ = 19%, *P*=0.29). Therefore, the fixed-effect model was adopted for meta-analysis. The meta-analysis results of the fixed-effect model showed that there was no significant difference between the Wenxin granules group and the propafenone group. The difference was not statistically significant (OR = 1.17, 95% CI (0.65, 2.11), *P*=0.60).

#### 3.4.4. Comparison 4: Wenxin Granules with Propafenone 100 mg

Two trials [19, 20] were involved in this comparison; there was no statistical heterogeneity among the studies (*I*^2^ = 0%, *P*=0.81). Therefore, the fixed-effect model was adopted for meta-analysis. The meta-analysis results of the fixed-effect model showed that the Wenxin granules group was better than the propafenone group. The difference was statistically significant (OR = 2.56, 95% CI (1.34, 4.89), *P*=0.004).

#### 3.4.5. Comparison 5: Wenxin granules with Propafenone 100–150 mg

One trial [18] was involved in this comparison; the results of the meta-analysis showed that there was no significant difference between the Wenxin granules group and the propafenone group. The difference was not statistically significant (OR = 2.01, 95% CI (0.79, 5.13), *P*=0.14).

**Figure 3:**
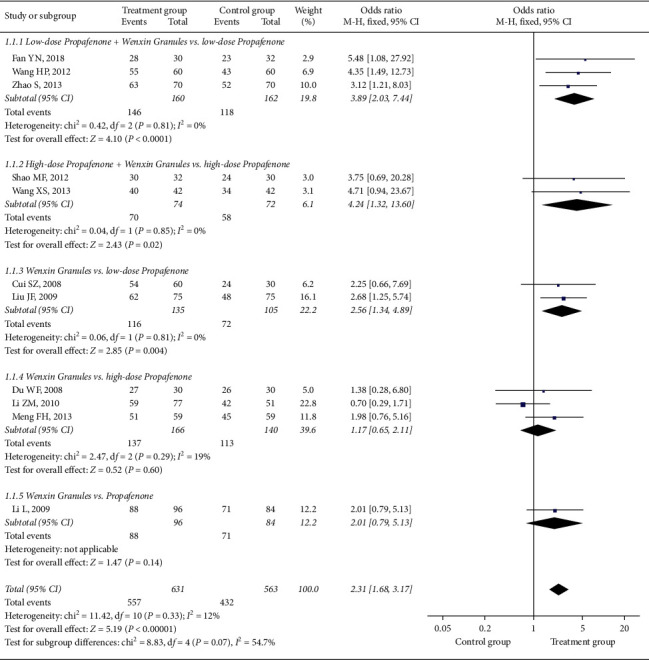
The forest plot of outcome measure clinical efficacy.

Subgroup analysis results showed that the statistical heterogeneity in different subgroups was low, and there was statistical heterogeneity between the groups (*I*^2^ = 54.7%) (see Figure 3).

#### 3.4.6. The Overall Quality of Evidence by GRADE

Four meta-analyses were conducted in this part; all four meta-analyses were conducted on the primary outcome indicator—clinical efficacy (details in Table 4). Therefore, Wenxin granules had a low quality of evidence on the clinical effective rate of treating atrial premature beats.

### 3.5. Adverse Effects Rate

A total of 9 studies using the incidence of adverse reactions as an indicator were included [4, 5, 12, 13, 16–18, 20]. Two studies [14, 19] did not mention specific adverse reactions. No significant adverse reactions occurred in 1 study [15].

**Figure 4:**
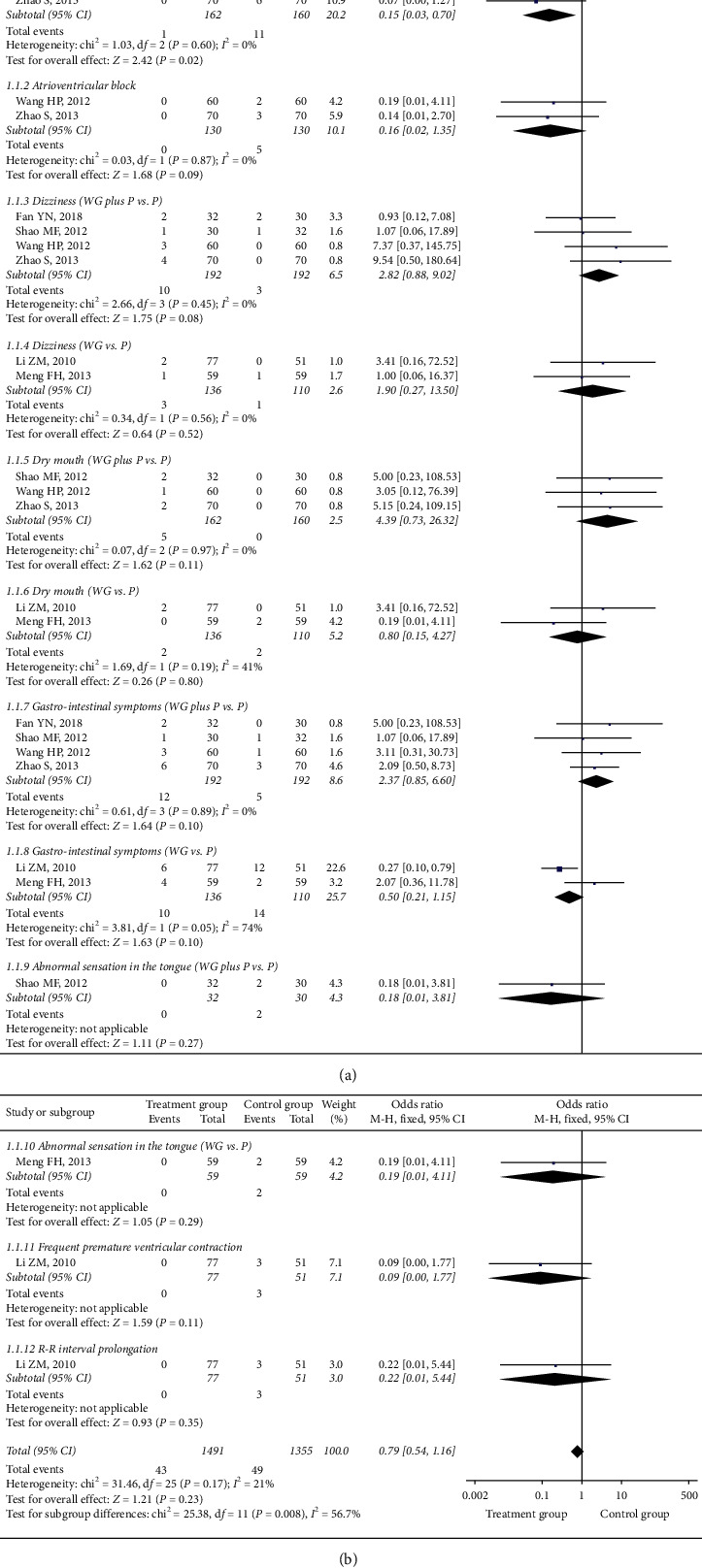
The forest plot of outcome measure adverse reactions.

The meta-analysis results of adverse reactions are shown in Table 5 and Figure 4.

## 4. Discussion

This study conducted a meta-analysis on the efficacy and safety of Wenxin granules and propafenone in the therapy of APB. We adopted strict inclusion and exclusion criteria for screening and included 11 studies (including 1194 patients). As there are two dosage types of Buchang Wenxin granules, 9 g (containing sugar) and 5 g (not containing sugar), and the dosages of its effective components are the same, it can be considered that there is no difference between them. Wang et al. [21] showed that Wenxin granules could prolong the sufficient refractory period of the myocardium by blocking sodium and potassium channels, inhibiting triggering activity by inhibiting late sodium current, and higher concentration can reduce the dispersion of transpolar repolarization. It was concluded that Wenxin granules were safe and less likely to cause arrhythmia. A survey of arrhythmias in Chinese hospitalized patients conducted by the Chinese Medical Association of Pacing and Electrophysiology and the Cardiovascular Prevention and Control Centre affiliated to the Ministry of Health in 2007 showed that Wenxin granules ranked sixth among all Traditional Chinese and Western Medicines for antiarrhythmic use. Wenxin granules were the most commonly used antiarrhythmic proprietary Chinese medicines used by Chinese people.

Meta-analysis results showed that Wenxin granules combined with propafenone have better clinical efficacy than the use of propafenone alone in the treatment of APB. There is no difference in clinical efficacy between the higher dose propafenone and Wenxin granules alone in the treatment of APB, and Wenxin granules alone are superior to the low-dose propafenone in the treatment of APB. Wenxin granules combined with propafenone can reduce the incidence of sinus bradycardia caused by propafenone. There is no significant difference between Wenxin granules combined with propafenone and propafenone alone in causing the atrioventricular block, dizziness, xerostomia, gastrointestinal symptoms, and tongue paresthesia. There is no significant difference between Wenxin granules alone and propafenone alone in causing dizziness, xerostomia, gastrointestinal symptoms, tongue paresthesia, frequent premature ventricular contractions, and prolongation of R-R interval.

## 5. Strengths and Limitations

We systematically conducted a comprehensive search of conference articles and unpublished literature registered on the clinical trial registry website. We tried to contact the author of the article to obtain more comprehensive information, but limited by the quality of included RCTs, the conclusions of this study still need further verification.

## 6. Implications for Research

Future clinical studies should include sufficient patients and use appropriately randomized, blinded, and statistical methods. We suggest that the following aspects should be paid attention to in future relevant randomized controlled trials: patients included should be diagnosed according to the latest international guidelines, with uniform standards and clear diagnosis, strict inclusion and exclusion criteria should be formulated, and age groups should be clearly distinguished. Baseline data and outcome index data of the treatment group and the control group should be completely described. We uniformly adopt the efficacy rating scale recommended by the latest international guidelines for further statistical analysis in the future. Sufficient attention should be paid to the follow-up of patients and the time should be long enough to observe the occurrence of long-term adverse reactions. Clinical studies should be registered in advance and eventually provide experimental data.

## 7. Conclusion

Very low-quality evidence showed that Wenxin granules may be superior to low-dose propafenone in the treatment of APB, and Wenxin granules may reduce the incidence of sinus bradycardia caused by propafenone. Therefore, Wenxin granules can be selected as one of the drug treatment schemes when clinically aiming at APB diseases, or Wenxin granules can be selected as a substitute drug for treatment when adverse drug reactions occur due to patients taking propafenone and normal quality of life is affected, so as to improve the living quality of patients and reduce the occurrence of adverse reactions. Limited by the quality of included RCTs, the conclusions of this study still need further verification.

## Figures and Tables

**Table 1:** The Latin and English names of the components for Wenxin granules.

Wenxin granules (product with code number approved by SFDA Z10950026)		
Traditional Chinese medicine name	Latin name	English name
GanSong	*Nardostachys chinensis* Bat.	Rhizoma Nardostachyos
DangShen	*Codonopsis pilosula* (Franch.) Nannf.	Lanceolata
SanQi	*Panax pseudoginseng* Wall. var. *notoginseng* (Burkill) Hoo and Tseng	Panax Notoginseng
HuPo	Succinum	Amber
HuangJing	*Polygonatum sibiricum* Delar. ex Redoute	Rhizoma Polygonatum

**Table 2:** Searching strategy for electronic databases.

Databases	Date
PubMed, and modified for the other two English databases	01/06/2019
#1. Wenxin granules [All Fields] OR Wenxin keli [All Fields]	
#2. atrial premature beats [All Fields] OR premature atrial contractions [All Fields] OR atrial premature complex [All Fields] OR atrial premature contraction [All Fields] OR premature atrial complex [All Fields] OR atrial extrasystole OR PAC [All Fields]	
#3. English [Language]	
#4. #1 AND #2 AND #3	

China National Knowledge Infrastructure (CNKI), and modified for the other two Chinese databases	01/06/2019
#1. Wenxin keli (Wenxin granules)	
#2. fangxingzaobo (atrial premature beats) OR fangxingqiqianshousuo (premature atrial contractions) OR fangzao (atrial extrasystole)	
#3. #1 AND #2	

**Table 3:** Characteristics of included RCTs on Wenxin granules and propafenone for treating atrial premature beats.

Study ID	Sample size	Sex (M/F)	APB duration (units: year)	Age (years, average or range)	Interventions			Outcomes
					T	C	Course of the treatment	
Shao MF 2012 [12]	T:32 C:30	T:15/17 C:14/16	T:0.03–12 (mean: 2.6) C:0.03–11.5 (mean: 2.4)	T:20–78 (Mean: 56) C:22–76 (Mean: 53)	Propafenone 150 mg Tid po and Wenxin granules 5 g Tid po	Propafenone 150 mg Tid po	4 weeks	①②
Fan YN 2018 [13]	T:30 C:32	T:19/11 C:20/12	NR	T:68.23 ± 12.29 C:66.18 ± 10.63	Propafenone 100 mg Tid po and Wenxin granules 5 g Tid po	Propafenone 100 mg Tid po	4 weeks	①②
Wang HP 2012 [5]	T:60 C:60	62/58	1–9	75.34 ± 4.32	Propafenone 100 mg Tid po and Wenxin granules 9 g Tid po	Propafenone 100 mg Tid po	4 weeks	①②
Zhao S 2013 [4]	T:70 C:70	71/69	NR	75.33 ± 4.31	Propafenone 100 mg Tid po and Wenxin granules 1 package Tid po	Propafenone 100 mg Tid po	4 weeks	①②
Wang XS 2013 [14]	T:42 C:42	T:23/19 C:22/20	0.25–10	T:48.12 ± 3.89 C:47.69 ± 3.54	Propafenone 150 mg Tid po and Wenxin granules 5 g Tid po	Propafenone 150 mg Tid po	4 weeks	①②
Du WF 2008 [15]	T:30 C:30	34/26	NR	>60 (Mean:73.5)	Wenxin granules 1 package Tid po	Propafenone 150 mg Tid po	4 weeks	①②
Li ZM 2010 [16]	T:77 C:51	T:48/29 C:32/19	NR	T:56.32 ± 19.65 C:55.19 ± 17.28	Wenxin granules 9 g Tid po	Propafenone 150 mg Tid po	4 weeks	①②
Meng FH 2013 [17]	T:59 C:59	T:36/23 C:35/24	NR	T:41–77 (Mean: 57) C:40–77 (Mean: 57)	Wenxin granules 9 g Tid po	Propafenone 150 mg Tid po	4 weeks	①②
Li L 2009 [18]	T:96 C:84	T:51/45 C:44/40	NR	T:Mean:63.2 C:Mean:60.9	Wenxin granules 9 g 2–3 times a day po	Propafenone 100–150 mg 2–3 times a day po	15–30 days	①
Cui SZ 2008 [19]	T:60 C:30	NR	NR	T:72.80 ± 12.40 C:70.60 ± 14.40	Wenxin granules 9 g Tid po	Propafenone 100 mg Tid po	4 weeks	①②
Liu JF 2009 [20]	T:75 C:75	T:40/35 C:36/39	NR	T:56.20 ± 5.40 C:54.60 ± 4.90	Wenxin granules 9 g Tid po	Propafenone 100 mg Tid po	4 weeks	①

Outcome: ①Clinical efficiency; ②Drug adverse reactions. ^#^T: treatment group; C: control group; M: males; F: females; NR: not reported; APBs: atrial premature beats.

**Table 4:** Summary of main findings of Wenxin granules and propafenone treating APB.

Wenxin granules (or plus propafenone) vs propafenone for APB					
Patient or population: Patients with APB settings: Outpatient or inpatient intervention: Wenxin granules (or plus propafenone) vs propafenone					

Outcomes	Illustrative comparative risks^*∗*^ (95% CI)		Relative effect (95% CI)	No of Participants (studies)	Quality of the evidence (GRADE)
	Assumed risk	Corresponding risk			
	Control	Wenxin granules vs propafenone			

Clinical efficacy: low-dose propafenone + Wenxin granules vs low-dose propafenone	728 per 1000	913 per 1000 (845 to 952)	OR 3.89 (2.03 to 7.44)	322 (3 studies)	⊕⊝⊝⊝ very low^1,2^

Clinical efficacy: high-dose propafenone + Wenxin granules vs high-dose propafenone	806 per 1000	946 per 1000 (845 to 983)	OR 4.24 (1.32 to 13.6)	146 (2 studies)	⊕⊝⊝⊝ very low^1,2^

Clinical efficacy: Wenxin granules vs low-dose propafenone	686 per 1000	848 per 1000 (745 to 914)	OR 2.56 (1.34 to 4.89)	240 (2 studies)	⊕⊝⊝⊝ very low^1,2^

Clinical efficacy: Wenxin granules vs high-dose propafenone	807 per 1000	830 per 1000 (731 to 898)	OR 1.17 (0.65 to 2.11)	306 (3 studies)	⊕⊝⊝⊝ very low^1,3^

Clinical efficacy: Wenxin granules vs propafenone	845 per 1000	917 per 1000 (812 to 966)	OR 2.01 (0.79 to 5.13)	180 (1 study)	⊕⊝⊝⊝ very low^1,2^

^*∗*^The basis for the assumed risk (e.g., the median control group risk across studies) is as follows: the corresponding risk (and its 95% confidence interval) is based on the assumed risk in the comparison group and the relative effect of the intervention (and its 95% CI). CI: confidence interval; OR: odds ratio; GRADE: working group grades of evidence. High quality: further research is very unlikely to change our confidence in the estimate of effect. Moderate quality: further research is likely to have an important impact on our confidence in the estimate of effect and may change the estimate. Low quality: further research is very likely to have an important impact on our confidence in the estimate of effect and is likely to change the estimate. Very low quality: we are very uncertain about the estimate. ^1^There were very serious limitations of methodological quality of included trials according to the risk of bias assessment. No explanation was provided. ^2^ There were very serious limitations of imprecision. The number of incidents was less than 200 or had a wide 95% confidence interval. ^3^ There were serious limitations of imprecision. The number of incidents was less than 400, or the 95% confidence interval contained 1.

**Table 5:** The adverse reactions of Wenxin granules and propafenone.

Adverse reactions	Study ID	Treatment group		Control group		Interventions	OR	95%CI
		Events	Total	Events	Total			
Sinus bradycardia	Shao MF2012	1	32	2	30	Wenxin granules and propafenone vs propafenone	0.45	[0.04,5.26]
	Wang HP2012	0	60	3	60	Wenxin granules and propafenone vs propafenone	0.14	[0.01,2.69]
	Zhao S2013	0	70	6	70	Wenxin granules and propafenone vs propafenone	0.07	[0.00,1.27]
	*I* ^2^ = 0% (*P*=0.60)					*P*=0.02	0.15	[0.03,0.70]

Atrioventricular block	Wang HP2012	0	60	2	60	Wenxin granules and propafenone vs propafenone	0.19	[0.01,4.11]
	Zhao S2013	0	70	3	70	Wenxin granules and propafenone vs propafenone	0.14	[0.01,2.70]
	*I* ^2^ = 0% (*P*=0.87)					*P*=0.09	0.16	[0.02,1.35]

Dizziness	Shao MF2012	2	32	2	30	Wenxin granules and propafenone vs propafenone	0.93	[0.12,7.08]
	Fan YN2018	1	30	1	32	Wenxin granules and propafenone vs propafenone	1.07	[0.06,17.89]
	Wang HP2012	3	60	0	60	Wenxin granules and propafenone vs propafenone	7.37	[0.37,145.75]
	Zhao S2013	4	70	0	70	Wenxin granules and propafenone vs propafenone	9.54	[0.50,180.64]
	*I* ^2^ = 0% (*P*=0.45)					*P*=0.33	1.64	[0.60,4.45]
	Li ZM2010	2	77	0	51	Wenxin granules vs propafenone	3.41	[0.16,72.52]
	Meng FH2013	1	59	1	59	Wenxin granules vs propafenone	1.00	[0.06,16.37]
	*I* ^2^ = 0% (*P*=0.56)					*P*=0.52	1.90	[0.27,13.50]

Dry mouth	Shao MF2012	2	32	0	30	Wenxin granules and propafenone vs propafenone	5.00	[0.23,108.53]
	Wang HP2012	1	60	0	60	Wenxin granules and propafenone vs propafenone	3.05	[0.12,76.39]
	Zhao S2013	2	70	0	70	Wenxin granules and propafenone vs propafenone	5.15	[0.24,109.15]
	*I* ^2^ = 0% (*P*=0.97)					*P*=0.11	4.39	[0.73,26.32]
	Li ZM2010	2	77	0	51	Wenxin granules vs propafenone	3.41	[0.16,72.52]
	Meng FH2013	0	59	2	59	Wenxin granules vs propafenone	0.19	[0.01,4.11]
	*I* ^2^ = 41% (*P*=0.19)					*P*=0.85	1.19	[0.19,7.63]

Gastrointestinal symptoms	Shao MF2012	2	32	0	30	Wenxin granules and propafenone vs propafenone	5.00	[0.23,108.53]
	Fan YN2018	1	30	1	32	Wenxin granules and propafenone vs propafenone	1.07	[0.06,17.89]
	Wang HP2012	3	60	1	60	Wenxin granules and propafenone vs propafenone	3.11	[0.31,30.73]
	Zhao S2013	6	70	3	70	Wenxin granules and propafenone vs propafenone	2.09	[0.50,8.73]
	*I* ^2^ = 0% (*P*=0.89)					*P*=0.10	2.37	[0.85,6.60]
	Li ZM2010	6	77	12	51	Wenxin granules vs propafenone	0.27	[0.10,0.79]
	Meng FH2013	4	59	2	59	Wenxin granules vs propafenone	2.07	[0.36,11.78]
	*I* ^2^ = 74% (*P*=0.05)					*P*=0.10	0.50	[0.21,1.15]

Abnormal sensation in the tongue	Shao MF2012	0	32	2	30	Wenxin granules and propafenone vs propafenone	0.18	[0.01,3.81]
	Not applicable					*P*=0.27	0.18	[0.01,3.81]
	Meng FH2013	0	59	2	59	Wenxin granules vs propafenone	0.19	[0.01,4.11]
	Not applicable					*P*=0.29	0.19	[0.01,4.11]

Frequent premature ventricular contraction	Li ZM2010	0	77	3	51	Wenxin granules vs propafenone	0.09	[0.00,1.77]
	Not applicable					*P*=0.11	0.09	[0.00,1.77]

R-R interval prolongation	Li ZM2010	0	77	1	51	Wenxin granules vs propafenone	0.22	[0.01,5.44]
	Not applicable					*P*=0.35	0.22	[0.01,5.44]

## Data Availability

The data used to support the findings of this study are available from the corresponding author upon request.
